# Case report: Fulminant extraneural metastasis of glioblastoma through venous sinus

**DOI:** 10.3389/fonc.2022.1034944

**Published:** 2022-10-21

**Authors:** Yeong Jin Kim, Kang Hee Ahn, Kyung-Hwa Lee, Kyung-Sub Moon

**Affiliations:** ^1^ Department of Neurosurgery, Chonnam National University Hwasun Hospital, Chonnam National University Medical School, Hwasun, Jeollanam-do, South Korea; ^2^ Department of Pathology, Chonnam National University Hwasun Hospital, Chonnam National University Medical School, Hwasun, Jeollanam-do, South Korea

**Keywords:** extracranial, extraneural, glioblastoma, metastasis, venous sinus

## Abstract

**Background:**

Extraneural metastasis (ENM) of glioblastoma are rare. However, as patient overall survival improves, the incidence of ENM has gradually increased. Although several risk factors have been proposed, venous sinus invasion was regarded as a very exceptional route for ENM.

**Case description:**

We report a 60-year-old man with glioblastoma in the temporal lobe, invading the transverse and sigmoid venous sinus. After gross total tumor resection, the patient received the standard chemoradiation therapy. Systemic evaluation for persistent shoulder and back pain revealed widespread metastasis to lymph nodes and multiple bones 9 months after surgery. Despite spine radiation therapy, the patient became paraplegic and died 1 year after surgery.

**Conclusions:**

Venous sinus invasion should be kept in mind by physicians, as a risk factor for glioblastoma ENM. Systemic evaluation of these patients with extracranial symptoms should be performed without hesitation.

## Introduction

Glioblastoma is the most common and fatal primary central nervous system (CNS) cancer with highly infiltrative growth. After maximal safe resection, concurrent chemoradiotherapy and adjuvant chemotherapy using temozolomide (TMZ) has been used as standard treatment for initially diagnosed glioblastoma. Despite the improvement of surgical intervention and various treatment strategies, the average survival period is around 15-18 months and 5-year survival rate was reported as 6.8% in the developed country (1).

Unlike most systemic cancers in other organs with high frequency of brain metastasis, the systemic extraneural metastasis (ENM) of glioblastoma was rare (0.4%-0.5% of all cases) possibly due to strong barriers formed by the dura mater and blood-brain barrier (BBB) (1, 2). However, in a systemic review for the published ENM cases, Pietschmann et al. reported a remarkable increase in the number of reported cases per decade over time (3). This increase was possibly related to rising awareness of the ENM along with improvement of imaging diagnosis. Since the majority of ENM developed at the late stage of clinical course with a median interval of 9 months or 2 years from the initial diagnosis (3, 4), the higher chance of ENM was also related to improved local tumor control and prolonged survival of glioblastoma. Additionally, although glioblastoma may be less common in Asian and African countries possibly due to differences in age distribution and diagnostic accessibility (1), the age-adjusted incidence rate of glioblastoma has recently increased in North American and European countries (5, 6). The improvement in diagnostic techniques, lifestyle changes, or environmental factors might be responsible for the increased incidence rate of glioblastoma, leading the increased chance of ENM (7, 8). Therefore, clinician should be aware of the possibility of ENM in glioblastoma and its risk factors to prevent oversight and misdiagnosis.

## Case presentation

**Figure 1:**
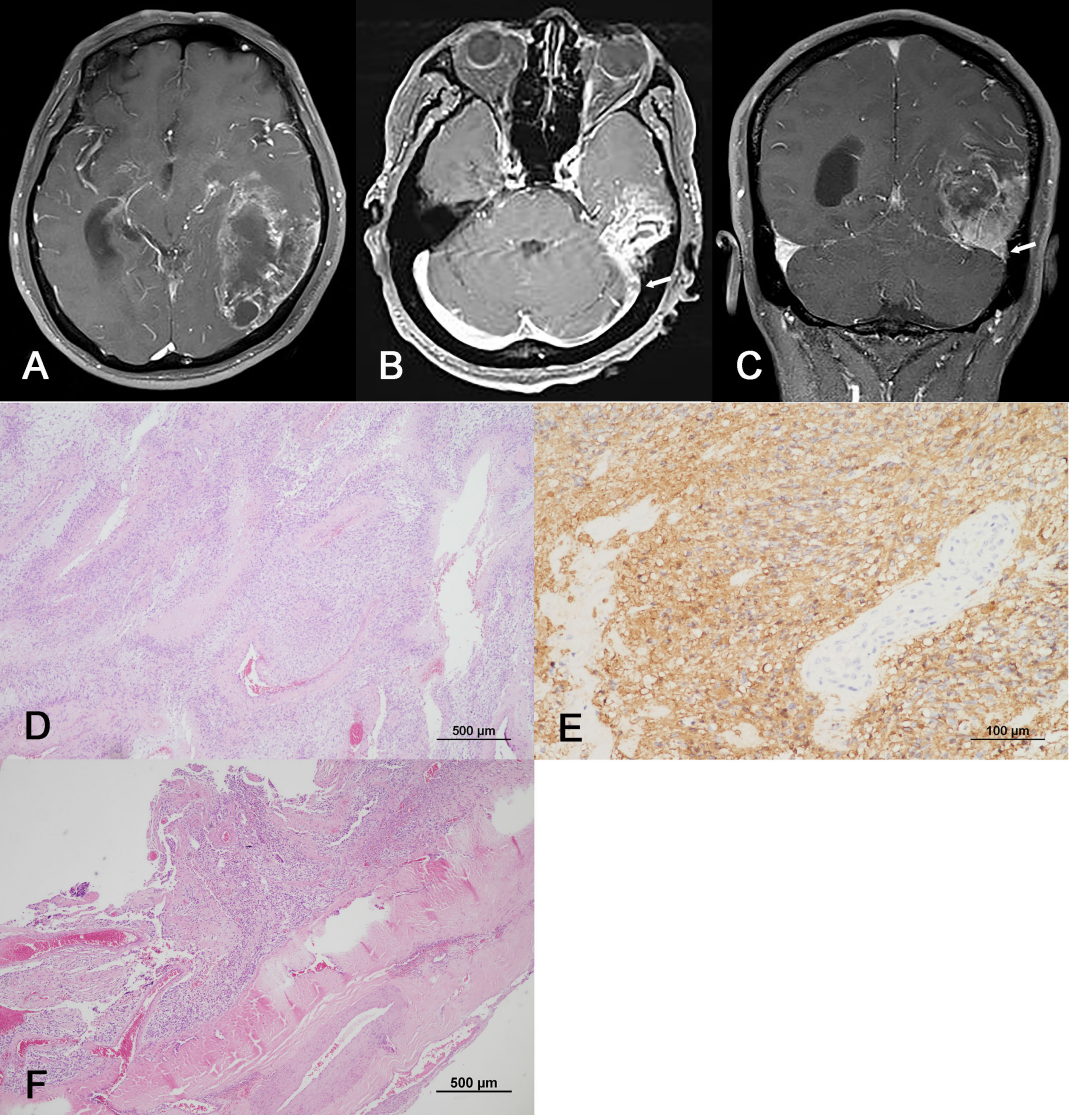
Radiological and pathological images of the initial mass. **(A–C)** Preoperative Gd-enhanced T1-weighted MRI showing a heterogeneously enhanced mass in the left temporal region with leptomeningeal involvement. Note the lesion invading the transverse and sigmoid sinuses (arrow head). **(D, E)** Representative microphotos of the primary surgical sample with palisading tumor cell necrosis and GFAP immunopositivity, consistent with a diagnosis of glioblastoma. **(F)** The removed dural tissue is involved by glioblastoma (**D, F**: hematoxylin and eosin staining, original magnification ×40, **(E)**: GFAP immunohistochemistry, original magnification ×200).

A 60-year-old man presented with a 2-month history of cognitive impairment. He had hypertension and a history of early gastric cancer surgery 10 years previously. There were no abnormal radiological findings on abdominopelvic or chest computed tomography (CT). Brain magnetic resonance imaging (MRI) showed a large contrast-enhancing (67×48×60 mm) lesion in the left temporal lobe, invading the transverse and sigmoid venous sinuses (**Figures 1A–C**).

**Figure 2:**
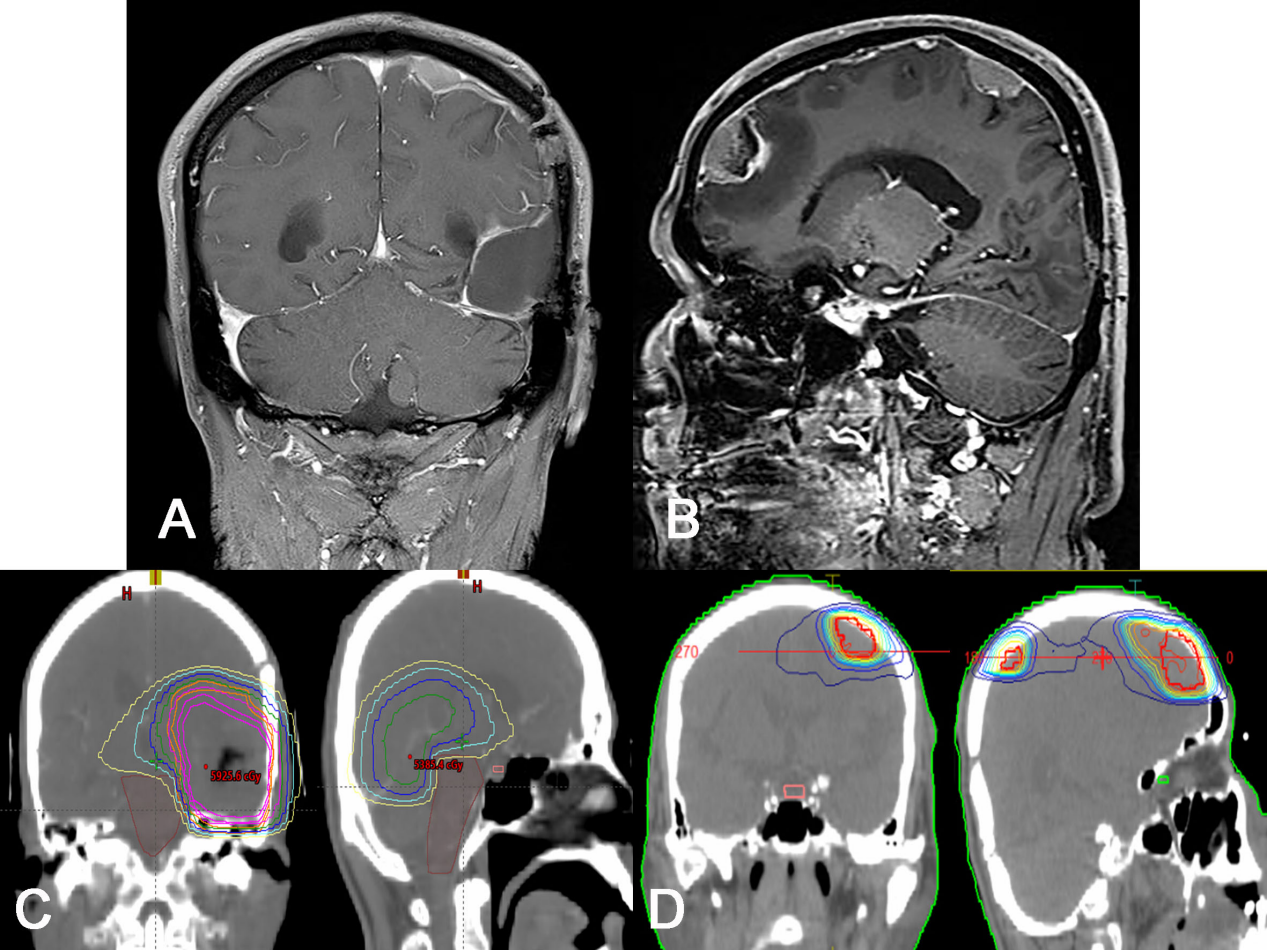
Radiological and treatment plan images for recurred lesions. **(A, B)** Gd-enhanced T1-weighted MRI showing two enhanced, dural based masses along the dura mater of remote site. Note that there is no definitive recurrence on the initial lesion. **(C, D)** Representative photos of dose plans for initial radiation (**C**, 60 Gy) and re-irradiation (**D**, 50 Gy) treatments. Note that there is no definitive overlap in radiation field in two dose plans.

Suspecting glioma, a craniotomy and gross total tumor resection were performed. At surgery, an infiltrating mass was found invading the dura mater, including a sinus wall component. The pathologic diagnosis was isocitrate dehydrogenase (IDH)-wildtype glioblastoma with O6-methylguanine-DNA methyltransferase (MGMT) promoter methylation. Tumor cell invasion of the dura was confirmed histopathologically (**Figures 1D–F**). The patient underwent concurrent chemoradiation therapy (60 Gy) and three cycles of adjuvant TMZ. Unfortunately, two remote meningeal enhancing lesions in the left frontal and parietal convexity were found on follow-up MRI 5 months after surgery (**Figures 2A, B**) and the patient was treated with metronomic TMZ plus radiation therapy (RT, 50 Gy). Since there was no overlap of the radiation fields compared to the initial RT plan, there was little possibility of radiation necrosis (**Figures 2C, D**). At that time, the patient had intermittent right shoulder and back pain. Chest and shoulder x-rays were performed, but there were no specific radiologic findings.

**Figure 3:**
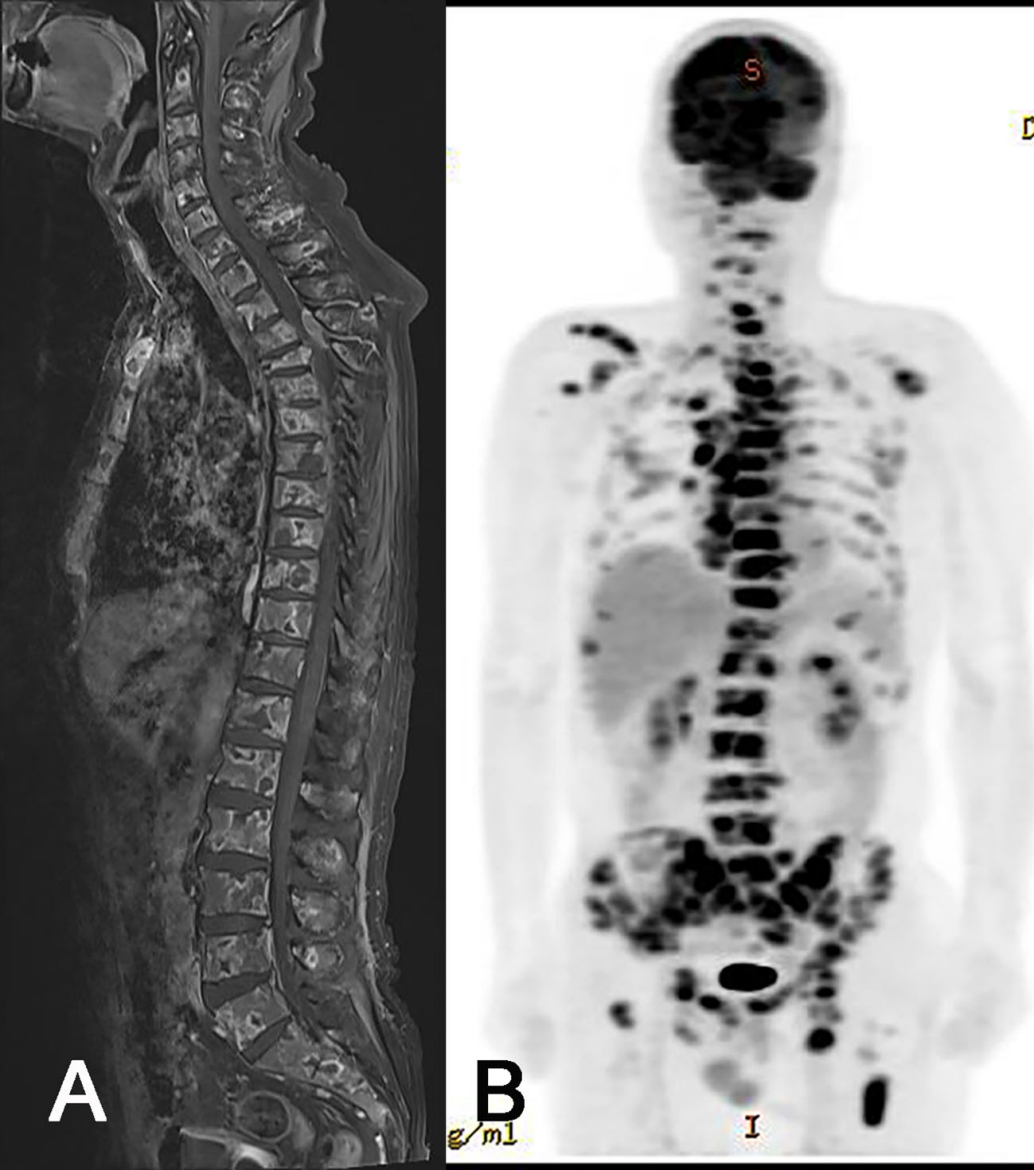
Radiologic images of extraneural metastasis (ENM). Whole spine MRI **(A)** and PET-CT **(B)** showing extensive ENM to multiple bones and lymph nodes.

**Figure 4:**
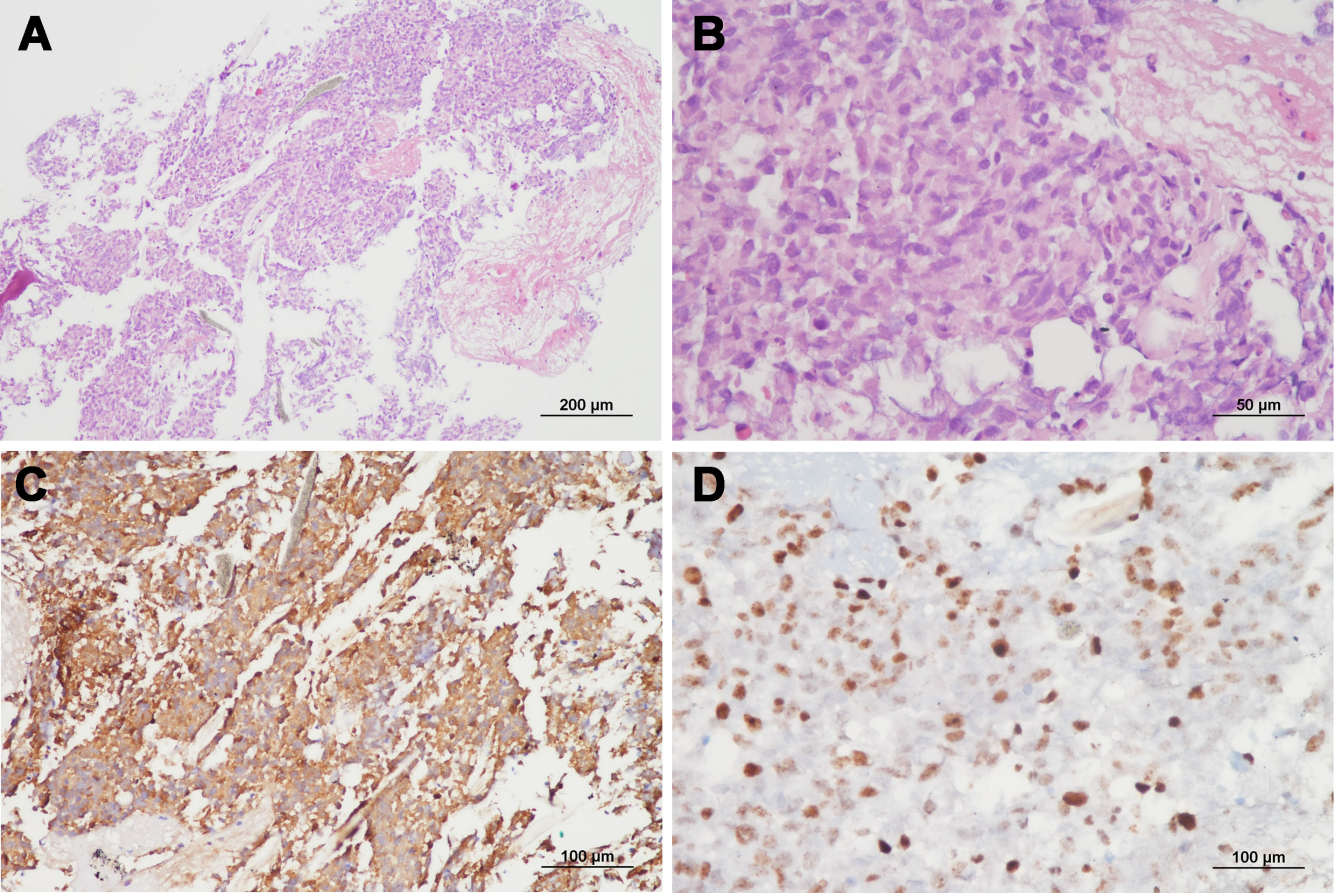
Representative microphotos of a vertebral lesion with GFAP immunopositivity and high Ki-67 proliferation index, consistent with a diagnosis of metastatic glioblastoma. (**A, B**: hematoxylin and eosin staining, original magnification ×100 & ×400, **C**: GFAP immunohistochemistry, original magnification ×200, **D**: Ki-67 staining, original magnification ×400).

Due to progressive shoulder and back pain, systemic evaluations including spine MRI, positon emission tomography (PET)-CT, and chest and abdomen CT were done 9 months after surgery and revealed widespread metastases to the chest & cervical lymph nodes and multiple bones, including the vertebrae, scapula, and pelvis (**Figure 3**). The largest vertebral lesion was biopsied and diagnosed as metastatic glioblastoma as evidenced by glial fibrillary acidic protein (GFAP) immunopositivity (**Figure 4**). Despite spine RT (24 Gy), the patients became paraplegic, was admitted to palliative care, and died 1 year after surgery.

## Discussion

Previous studies reported that risk factors for glioblastoma ENM include invasive procedures, RT, young age, prolonged survival time, tumor recurrence, and sarcomatous component (3, 9). Also, ENM occurred much more frequently in males, and the most common tumor location was in the temporal lobe (3, 10). Generally, chemoradiation therapy removes most of the tumors, but can promote the selective evolution of treatment-resistant tumor cell clones, which can result in more aggressive biological behavior. Surgery and RT can also promote ENM by breaking the tight junction of the BBB. As a result, tumor treatment can play a role in helping metastasis to occur more easily. However, about 10% of ENMs occur in patients who have not undergone surgery and there was no significant difference in the circulating tumor cell numbers in patients before or after surgery. Even before treatment, about 20% of glioblastoma patients had circulating tumor cells in the peripheral blood (11). As metastatic precursor cells, circulating tumor cells have more mesenchymal phenotype and stem cell like properties (12). The therapeutic approach may not be an essential factor for metastases and its role in ENM remains to be demonstrated.

Glioblastoma ENM probably have a histopathological and/or genetic predisposition. To develop distant metastases in systemic cancers, epithelial-mesenchymal transition (EMT) can be required. Sarcomatous metaplasia in glioblastoma is an example of EMT. With a sarcomatous phenotype, glioblastoma cells can degrade extracellular matrix proteins, promoting vascular or lymphatic invasion. Some tumor clones have some specific genotypes that predisposes to ENM. In analysis of genetic predisposition, glioblastoma with ENM had a tendency of tumor suppressor gene alteration. Common genomic alteration included TP53, ATRX, PTEN, RB1, TERT, IDH1, and NF1 (13). Among them, TP53 mutation has been most frequently reported mutation (13–16). As p53 plays an important role in DNA repair, TP53 mutation could induce repertory of another genetic mutation that make ENM possible. An aggressive sarcomatous phenotype or other unknown genotype might promote vascular or lymphatic invasion and systemic dissemination of glioblastoma.

Considering common metastatic sites (lung, bone, and lymph node) and wide dissemination feature in ENM, the metastatic route is probably *via* a vascular or lymphatic pathway (3). In this case, the tumor invaded the venous sinus and surrounding dura mater, and tumor cells were likely shed into the venous blood and circulating blood flow. Since the widespread ENM through the superior sagittal sinus was proposed in postmortem examination for an untreated malignant astrocytoma (17), previous studies showed that venous sinus invasion is a risk factor for metastases in glioblastoma (18, 19, **Table 1**). In addition, lymphatic vessels in the meninges are a network of narrow channels that run mostly along the sagittal, transverse, and sigmoid venous sinuses. The lymphatic network at the base of the brain is dense and exits the skull along with the cranial nerves (20). In a tumor invading the dura mater around a venous sinus, tumor cells can enter the lymphatic circulation. In this case, the tumor recurrence was along the dura mater without definitive recurrence at the initial site, and there was multiple metastasis at cervical lymph nodes. These findings demonstrate the possibility of ENM along the lymphatic system.

**Table 1:** Summary of recently reported glioblastoma cases with ENM through venous sinus.

Case No. (ref.)	Age/Sex	GBM location	Involved sinus	Tx for primary GBM	Interval from GBM to ENM (mos)	ENM sites	Tx after ENM	Interval from GBM to death (mos)	Interval from ENM to death (mos)
1 (18)	51/Male	Left P	SSS	Radiochemotherapy	24*	Lung, pleura, rib	Radiation & chemotherapy with TMZ	25	1
2 (18)	30/Female	Left F	SSS	Radiochemotherapy	7^!^	Temporalis muscle, L5 body	Re-irradiation & chemotherapy with BEV+IRR	28	21
3 (19)	29/Female	Right T-P	TS	Radiochemotherapy, Re-irradiation for recurred lesion	22	Lung	Chemotherapy with BEV and carboplatin, VP shunt	35	13
4 (Our case)	60/Male	Left T-P	TS & SS	Radiochemotherapy, Re-irradiation & metronomic TMZ for recurred lesion	9	Multiple lymph nodes & bones	Palliative radiation treatment	12	3

BEV, bevacizumab; ENM, extraneural metastasis; F, frontal; GBM, glioblastoma; IRR, irrinotecan; mos, months; P, parietal; SS, sigmoid sinus; SSS, superior sagittal sinus; T-P, temporoparietal; TMZ, temozolomide; TS, transverse sinus; Tx, Treatment; VP, ventriculoperitoneal.

*From diagnosis of glioblastoma initially diagnosed as mixed glioma 5 years ago.

!L5 body metastasis was detected 25 months after initial glioblastoma.

The prognosis of glioblastoma with ENM is dismal. Median overall survival from diagnosis of ENM ranged from 1.5 months to 6 months. Although there was no prognostic significance in age, gender, initial tumor location, or time interval from initial diagnosis of primary tumor to detection of ENM, the presence of sarcomatous component and lung metastasis were possible poor prognostic factors (3, 13, 21). Because of the rarity and unknown pathogenesis of ENM, the treatment strategy for EMN has not been fully established. However, considering that ENM occurs frequently in young patients around the age of 40 (3, 13, 21), it is possible to combine targeted therapy (13, 16) or immunotherapy (22) with conventional treatment strategies (3, 21). Our patient had a glioblastoma with venous sinus invasion, a history of craniotomy and RT, and intracranial tumor recurrence at remote site. Unfortunately, although the patient had multiple risk factors and complained of pain due to ENM, a systemic evaluation was delayed.

## Conclusion

Venous sinus invasion should be considered as a possible route for ENM in glioblastoma. In managing patients with such risk factor, clinicians should not hesitate to perform a systemic evaluation when patients have extraneural symptoms.

## Data availability statement

The raw data supporting the conclusions of this article will be made available by the authors, without undue reservation.

## Ethics statement

Ethical review and approval were waived for this study, due to the fact that this is a single case report. Written informed consent was also obtained from the participant for the publication of this manuscript, including any potentially-identifying images or data. The patients/participants provided their written informed consent to participate in this study. Written informed consent was obtained from the individual(s) for the publication of any potentially identifiable images or data included in this article.

## Author contributions

Conceptualization: K-HL & K-SM. Data curation: KA, YK, and K-SM. Analysis and interpretation of data: K-HL & K-SM. Funding acquisition: K-SM. Writing-original draft: KA, YK, and K-SM. Writing-review & editing: K-HL. Manuscript approval: all authors. All authors contributed to the article and approved the submitted version.

## Funding

This work was supported by Basic Science Research Program through the National Research Foundation of Korea (NRF) funded by the Minist (2020R1C1C1007832 for K-SM, 2022R1A2C1011889 for K-HL). There was no role of the funding bodies in the design of the study, in the collection, analysis, and interpretation of data, or in the writing of the manuscript.

## Acknowledgments

The English in this document has been checked by at least two professional editors, both native speakers of English. For a certificate, please see: (http://www.textcheck.com/certificate/qoCxX6).

## Conflict of interest

The authors declare that the research was conducted in the absence of any commercial or financial relationships that could be construed as a potential conflict of interest.

## Publisher’s note

All claims expressed in this article are solely those of the authors and do not necessarily represent those of their affiliated organizations, or those of the publisher, the editors and the reviewers. Any product that may be evaluated in this article, or claim that may be made by its manufacturer, is not guaranteed or endorsed by the publisher.

## Abbreviations

BBB, Blood-brain barrier; CT, Computed tomography; ENM, Extraneural metastasis; MRI, Mmagnetic resonance imaging; TMZ, Temozolomide.
